# Case Report of Post-Appendectomy Fungal Osteomyelitis: A Rare Complication in a Healthy Patient

**DOI:** 10.5811/cpcem.35473

**Published:** 2025-02-15

**Authors:** Cameron Juybari, Andras Muranyi, Emmelyn J. Samones, Mindi Guptill

**Affiliations:** * Loma Linda University Medical Center, Department of Emergency Medicine, Loma Linda, California; † Loma Linda University Health, School of Medicine, Loma Linda, California

**Keywords:** Fungal osteomyelitis, appendectomy, Coccidioides, hematogenous osteomyelitis, case report

## Abstract

**Introduction:**

Osteomyelitis is a bone infection that presents with swelling, erythema, pain, and possible systemic symptoms. Immunocompromised patients are at higher risk of developing osteomyelitis. Fungal pathogens are a rare etiology for these infections with very few case reports published compared to infections due to bacterial pathogens. Work up should include imaging studies to investigate infections when there is clinical suspicion for osteomyelitis. Bone biopsy is performed to identify the causative agent with bacterial infections being the most common. Osteomyelitis can be treated both surgically with debridement or amputation and medically with extended courses of antimicrobials or antifungals. Our case describes fungal foot osteomyelitis after an uncomplicated appendectomy.

**Case Report:**

A 19-year-old previously healthy female underwent laparoscopic appendectomy for nonperforated, non-gangrenous appendicitis. Fourteen days later, she developed gradually worsening right foot pain, swelling, and erythema. After multiple failed treatments for the management of osteomyelitis, bone biopsies and courses of antibiotics, patient was ultimately diagnosed with a rare osteomyelitis secondary to *Coccidioides* species, which was managed and improved with antifungals.

**Conclusion:**

Bacterial osteomyelitis has been described in two English case reports as a postoperative complication of appendectomy, particularly when the appendicitis is perforated, gangrenous, or purulent. Fungal osteomyelitis is an even rarer cause of postoperative bone infection in immunocompetent patients. The goal for treatment is surgical intervention or pharmacologic management. Emergency department physicians should maintain a high suspicion for fungal osteomyelitis when otherwise healthy patients present multiple times for failing outpatient antibiotic regimens.

## INTRODUCTION

Osteomyelitis is an acute or chronic infection of bone tissue which occurs secondary to traumatic injury, open fractures, surgery or pyogenic organisms that spread through the bloodstream.^1^ Patients with a history of smoking cigarettes or diabetes, complicated and open fractures, surgical implants, large wound sites, and other causes for poor wound healing have increased risk for osteomyelitis. Osteomyelitis can present with inflammation, swelling, erythema at the site of infection and associated constitutional symptoms such as fever, chills, and malaise. Staphylococcus aureus is the most common bacterial infectious agent in osteomyelitis in adults and children; however, osteomyelitis can be caused by a variety of other bacterial or even fungal pathogens. Radiographs and magnetic resonance imaging (MRI) are standard methods of identification to detect soft tissue swelling, osteolysis, and other infection-related bone damage. Diagnostic criteria include positive cultures from bone or blood in conjunction with other signs and symptoms.^2^ Bone biopsy is generally performed to identify the infectious pathogen. Bone biopsy cultures identify microbiological cause in 94% of cases.^3^ Blood cultures are commonly negative except in cases in which osteomyelitis is caused by hematological spread.^4^ Treatment involves a multi-disciplinary approach with medical and surgical specialists for prolonged antibiotic therapy, and possible surgical debridement or amputation.^5^

This case of foot fungal osteomyelitis is unique presenting in a healthy patient after uncomplicated appendectomy. There is a paucity of research on the topic of osteomyelitis after appendectomy with only two case reports found in the English language literature review. The reported cases had bacterial pathogens as the causative agent and were in patients with ruptured, perforated, or gangrenous appendicitis.^6^,^7^

## CASE REPORT

A 19-year-old female with no significant past medical history presented to the Emergency department (ED) for progressively worsening right foot pain, swelling, and redness with associated nausea. The patient initially presented to the ED one month prior for abdominal pain, received pre-operative intravenous (IV) piperacillin/tazobactam, and underwent a laparoscopic appendectomy with findings of acute nonperforated appendicitis. She was discharged the same day with pain medications.

The patient followed up with the acute care surgery team in clinic on postoperative day 14. At the visit, she was determined to be doing well post-operatively. She expressed concern about swelling and soreness of her right foot without any history of trauma. Her physical exam noted that she was tender to palpation over the lateral right foot without any redness, wounds, bruising or any physical exam findings of trauma. The surgeon noted patient was able to bear weight and advised conservative management for possible foot sprain.

**Image 1:**
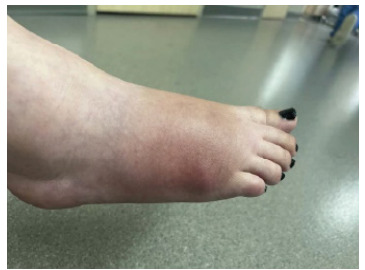
Patient with right foot fungal osteomyelitis after an appendectomy noted to have swelling, erythema, and tenderness to palpation on exam.

One month after the surgery, she presented to the ED because of persistent pain and swelling in the right foot. Initial vital signs were unremarkable. On physical exam, patient’s right lateral foot noted swelling with overlying erythema, no drainage, tender to palpitation and the distal foot was neurovascularly intact (Image 1), but no appreciable tenderness to palpation. Capillary refill was brisk, and radial pulses were 2+ and symmetric. He was noted to have erythema to the left forearm that had been present since birth. Radiographs of the right humerus and forearm were unremarkable. Ultrasound of the left upper extremity was obtained due to concern for thrombosis and revealed a long non-occlusive thrombus within the left axillary artery measuring approximately one centimeter in length, as well as a few small, non-occlusive adherent thrombi anteriorly and posteriorly in the midportion of the subclavian artery, with a distal area of turbulent arterial flow favored to be a thrombus.

Hematology was consulted and they made imaging recommendations. A magnetic resonance imaging (MRI) of the brain demonstrated a large acute/subacute infarct involving the right middle cerebral artery (MCA) and anterior cerebral artery with significant diffusion reduction. A magnetic resonance angiogram of the brain demonstrated severe focal occlusion at the M1/M2 junction of the MCA with normal flow seen in the distal MCA branches. Magnetic resonance venography of the brain was normal (Image 1).

**CPC-EM Capsule**

What do we already know about this clinical entity?

*Osteomyelitis is an infection of bone that can occur after surgery. It is rarely caused by fungal species*.

What makes this presentation of disease reportable?

*This case report highlights an unusual presentation of fungal osteomyelitis after uncomplicated appendectomy*.

What is the major learning point?

*If a patient continues to worsen in their treatment of osteomyelitis despite initial antibiotics, an atypical infection should be considered*.

How might this improve emergency medicine practice?

*This article encourages clinicians to expand their differential of bony pain after surgery and consider atypical infections for patients who failed conventional osteomyelitis treatment*.

She reported the symptoms started after her appendectomy. Significant labs include white blood cells (WBC), 8.18 x 109 per liter (L) (reference range: 4.8–11.80 x 109/L); erythrocyte sedimentation rate (ESR), 39 millimeters per hour (mm/hr) (0–20 mm/hr); C-reactive protein (CRP), 0.7 milligrams per deciliter (mg/dL) (0.0–0.8 mg/dL). Foot radiographs were concerning for osteomyelitis. Intravenous vancomycin was started in the ED, and she was admitted to the hospital. She had further radiologic studies. Computed tomography scan revealed findings suggestive of osteomyelitis with adjacent phlegmon and inflammatory changes and associated fifth metatarsal neck fracture. Magnetic resonance imaging reported findings consistent with osteomyelitis of the fifth metatarsal with surrounding phlegmon. Bone biopsy was negative to any growth.

Infectious disease was consulted and recommended IV cefazolin, thus a peripherally inserted central catheter (PICC) line was placed. She was discharged from the hospital with the diagnoses of osteomyelitis of the right fifth metatarsal and provided with a four-week course of cefazolin to be administered via her PICC line.

**Image 2:**
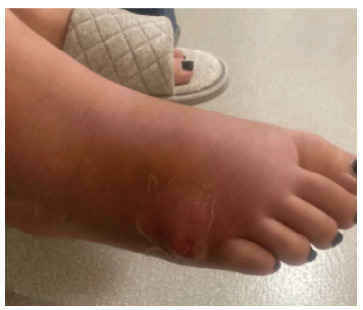
Patient with right foot fungal osteomyelitis after an appendectomy following a four-week course of cefazolin. She was noted to have worsening right foot pain, swelling, redness, with a new blister forming on exam.

Eight days later, patient returned to the ED for worsening right foot pain, swelling, redness, and a new blister forming on lateral side of foot. Vital signs remained unremarkable. Her physical exam was significant for swelling, tenderness, and redness with a fluctuant ulcer draining serous fluid (Image 2). Despite cefazolin, the patient’s symptoms had worsened.

Laboratory analysis revealed WBC, 7.05 x 109/L; ESR, 50 mm/hr; and CRP, 1.2 mg/dL. Acute care surgery was consulted for possible surgical debridement. Their team recommended internal medicine admission for IV antibiotics due to outpatient failure of cefazolin as well as infectious disease for the management of osteomyelitis. The patient was started on IV vancomycin and cefepime. Repeat MRI during this admission revealed worsening extensive osteomyelitis with regional phlegmon, developing abscess, myositis of the lateral forefoot and associated regional cellulitis. A second bone biopsy was performed and again had negative growth. Bone biopsy pathology reported marrow fibrosis with reactive changes but no acute inflammation or necrosis. Acute care surgery offered incision and drainage of the abscess and fifth digit amputation for source control which patient declined. As patient was deemed to have failed outpatient IV cefazolin therapy, infectious disease recommended four weeks of IV vancomycin and cefepime to be administered for four weeks via PICC line. Patient was discharged home with the regimen of antibiotics recommended by infectious disease.

**Image 3:**
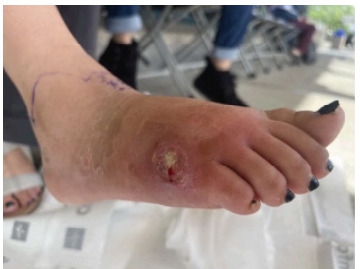
Patient with fungal osteomyelitis after an appendectomy following one month of cefepime and vancomycin. She was noted to have erythema of her right lateral foot with tenderness to palpation and a three-centimeter circular wound with purulent drainage on exam.

Three months after her appendectomy and one month after the second hospitalization, the patient again presented to the ED. During the antibiotic course with vancomycin and cefepime, she did experience temporary improvement but not resolution of her symptoms. Briefly after completion of the course, her symptoms returned. She presented with worsening pain and swelling of the right foot. On physical exam, she was noted to have erythema of her right lateral foot with tenderness to palpation and a three centimeter circular wound with purulent drainage (Image 3).

Laboratory analysis revealed WBC, 7.36 x 109/L; ESR, 53 mm/hr; and CRP, 1.9 mg/dL. Radiograph of the foot was suggestive of acute osteomyelitis with persistent phlegmon. She was started on IV cefazolin and admitted to the hospital under the internal medicine service. During that admission, her wound culture grew filamentous fungi and fungal specific culture to *Coccidioides immitis* and C posadasii. Infectious disease recommended voriconazole by mouth and discharge.

Upon follow up in the infectious disease clinic, the patient’s antifungal therapy was changed to fluconazole instead of voriconazole because of the side effects the patient experienced to voriconazole. As of writing this paper, the patient continues to take fluconazole for her improving foot infection.

After the patient’s appendectomy and multiple admissions to the hospital, the patient continues to follow up in clinic. The infectious disease team documented they theorize the patient may have acquired an asymptomatic Coccidioides pulmonary infection as she lives in an endemic area with dissemination to the foot because of an immunosuppressive event from her appendectomy. It is possible the patient’s osteomyelitis was autoimmune (chronic recurrent multifocal osteomyelitis) versus a secondary infection developing after multiple rounds of antibiotics, or related to skin breakdown, however a high suspicion for atypical fungal infection is critical in any patient diagnosed with osteomyelitis who does not improve with antibiotics.

## DISCUSSION

Bacterial osteomyelitis after appendectomy is a rare complication that has been reported in only two case reports.^6^,^7^ Fungal osteomyelitis is a rarer form of osteomyelitis with only case reports, case series, and narrative reviews in the published literature. From a recent (2023) systematic review of reported cases, the most common fungal pathogens in osteomyelitis are *Aspergillus* (26.5%), *Candida* (20.7%), and *Mucor* (16.8%) with *Coccidioides* osteomyelitis making up 5.6% of total cases. Vertebral fungal osteomyelitis is the most common (318 cases, 29.7%) while fungal foot osteomyelitis is rare (138 cases, 4.5%) and only 24 patients grew *Coccidioides* as the etiologic agent.^8^,^9^ Our patient is the only known reported case of fungal osteomyelitis after uncomplicated appendectomy.

The first case of osteomyelitis after an appendectomy reported in the English literature was published in 2010. The patient was a previously healthy male who developed appendicitis. He underwent appendectomy and was found to have a perforated retrocecal gangrenous appendix with copious free pus. He had an uncomplicated hospital course involving surgical site infection that required additional antibiotics. The patient re-presented six weeks after discharge with symptoms of right lower quadrant pain, and inability to bear weight. He was diagnosed with iliac crest osteomyelitis caused by *Pseudomonas aeruginosa* and *Bacteroides*.^6^

In 2013, the only other reported case of osteomyelitis following appendectomy involved a patient with a perforated gangrenous appendix with free pus. On postoperative day 23, he presented with lower back, thigh, and buttock pain and difficulty weight bearing. He was found to have sacroiliitis and iliac bone osteomyelitis which improved with antibiotic administration.^7^

Fungal osteomyelitis is considered a rare disease and is often overlooked when creating the differential diagnosis. Symptoms are often subacute and mimic those of other etiologies, which leads to substantial delays in treatment as was the case with our patient.^8^ Clinicians should consider fungal infection when osteomyelitis is of clinical concern. A recent (2023) systematic review of fungal osteomyelitis revealed the risk factors to be local surgery or local lesion, diabetes mellitus and disseminated fungal infection (which is theorized in our patient). However, fungal osteomyelitis can happen in an otherwise healthy patient as well.^9^

## CONCLUSION

Fungal osteomyelitis in the foot is rare and may present in an otherwise healthy patient but more commonly occurs in the patient with local surgery, local lesion, diabetes mellitus or disseminated fungal infection. It is critical to keep a broad differential diagnosis on postoperative patients presenting to the ED with musculoskeletal complaints. Clinicians should maintain high clinical suspicion for fungal infections when considering osteomyelitis especially for those who have risk factors and fail to improve after appropriate antibiotic courses.
